# The Contribution of CD40/CD40L Axis in Inflammatory Bowel Disease: An Update

**DOI:** 10.3389/fimmu.2015.00529

**Published:** 2015-10-16

**Authors:** Nezha Senhaji, Kevin Kojok, Youssef Darif, Christophe Fadainia, Younes Zaid

**Affiliations:** ^1^Laboratory of Genetic and Molecular Pathology (LGPM), Medical School, Hassan II University, Casablanca, Morocco; ^2^Laboratory of Thrombosis and Hemostasis, Montreal Heart Institute, Montreal, QC, Canada; ^3^Laboratory of Physiology and Molecular Genetic, Faculty of Sciences, Hassan II University, Casablanca, Morocco

**Keywords:** CD40, CD40L, inflammatory bowel disease, signaling pathways, immunity

## Abstract

Inflammatory bowel disease (IBD) is a chronic and multifactorial disease of the gastrointestinal tract. The exact etiology of IBD remains complex and unclear involving an inadequately defined relationship between microbial insult, genetic predisposition, altered intestinal barrier permeability, oxidative stress components and abnormal immune responses. The role of the co-stimulatory system made up of cluster of differentiation 40 protein (CD40) and its ligand (CD40L) in the response of the immune system to pathogens is now widely accepted. The implication of CD40/CD40L axis in immune system disorders due to its important role as signal transduction pathway among immune cells is well documented. Several studies have suggested that CD40/CD40L interactions regulate oxidative stress; this can affect various signaling pathways leading to IBD development. Hence, CD40/CD40L signaling pathway may become a new target for IBD treatment. This review will cover the general contribution of the CD40/CD40L dyad in the development of IBD in order to facilitate future approaches aiming to elucidate the immunological mechanisms that control gut inflammation.

## Inflammatory Bowel Disease

Inflammatory bowel diseases (IBDs) are chronic inflammatory disorder of the bowel. Their complex, multifactorial etiology remains unclear. Currently, the common assumption to explain the development of IBD would be a dysregulation of the mucosal immune response against intestinal flora elements, occurring in genetically predisposed patients (1). Crohn’s disease (CD) and ulcerative colitis (UC) are collectively referred to as IBD due to their common symptoms, characterized by evolution in spurts, interspersed with periods of remission. These two forms have an increasing severity and intensity over time and have important anatomical and pathological distinctions in particular regarding the extent and depth of the damage. The pathogenesis of these IBD entities results from a complex interaction of genetic, environmental, immunologic, and oxidative stress components, primarily driven by defects of the mucosal barrier (2, 3). They are characterized by recurring inflammation of the small and large intestine. Infiltration of macrophages, T and B lymphocytes into the intestinal epithelium disrupts its barrier function, resulting in diarrhea, abdominal pain, rectal bleeding, and malnutrition in attained individuals (4). Inflammatory lesions are secondary to an inappropriate and exaggerated immune response up to the entire bowel wall as in CD or confined to the surface layers as in UC. Inflammation in CD is characterized by the predominant production of Th1/Th17 type cytokines, such as IFN-γ, IL-12, and IL-17 (5, 6). While inflammation in UC is characterized by production of Th2-type cytokines, such as IL-13, IL-4, and IL-5 (5, 7). In addition, this incongruous activation of the immune system will lead to a perturbation of the immune balance that is necessary to maintain intestinal homeostasis.

Given its central role as a key regulator and amplifier of immune reactivity, the CD40/CD40 ligand (CD40L) system significantly contributes to the development and progression of multiple autoimmune diseases, including IBD. The present review discusses the latest observations relevant to the role of the CD40/CD40L pathway in the development of IBD.

## The CD40/CD40L Axis

Since its initial discovery more than two decades ago, the CD40/CD40L dyad has gained much attention in the scientific community. The pivotal role of CD40/CD40L dyad in immunity was initially evidenced by the finding that patients suffering from the X-linked hyper-IgM syndrome (HIGM) are characterized by mutations in their CD40L gene, resulting in loss of function in the CD40L protein. The resulting inactive CD40L protein is unable to induce T cell-dependent B cell responses, which are characterized by severe defects in humoral immunity as well as the absence of IgG, IgA, and IgE antibodies due to a lack of B cell Ig isotype switching (8). Now, it is clear that aside from its importance for appropriate immune responses, the CD40/CD40L dyad has a much broader cell expression pattern (Table S1 in Supplementary Materials) and it is associated with diverse physiological and pathological processes (Table S2 in Supplementary Materials).

A noteworthy role of CD40/CD40L in oxidative stress was first demonstrated in murine B lymphocytes, where increased production of reactive oxygen intermediates upon CD40 ligation correlated with c-Jun N-terminal kinase activation and IL-6 secretion (9). Moreover, activation of CD40 receptor increases oxidative stress by enhancing formation of endogenous ROS and as consequence inhibits endothelial cell migration (10). This role was further confirmed in mouse models where CD40 or CD40L deficiency abrogated hypercholesterolemia-induced oxidative stress (11).

CD40 is a member of the tumor necrosis factor receptor (TNFR) superfamily, which is constitutively or inducibly expressed on the surface of a variety of immune and non-immune cell types including B cells, macrophages, dendritic cells (DCs), microglia, endothelial cells, epithelial cells, and keratinocytes (12, 13). It was initially identified on B lymphocytes by antibody binding, then cloned in the Burkitt lymphoma Raji cell line, revealing a cDNA encoding for a 1.5 kb mRNA (14, 15).

Its ligand, CD40L (also known as CD154, gp39, TBAM, and TRAP), is a type II transmembrane protein belonging to the TNF superfamily (16) and is transiently expressed on the surface of activated CD4^+^ T cells, but can also be up-regulated on other cell types in the context of autoimmune disease (17, 18). The CD40–CD40L interaction results in movement of CD40 into cholesterol-rich membrane microdomains and the binding of TNFR-associated factors (TRAFs) to its cytoplasmic tail (12, 19). Specific TRAF molecules are associated with overlapping and distinct CD40-mediated functions (19). CD40 directly binds TRAF2, TRAF3, TRAF5, and TRAF6, and indirectly associates with TRAF1 (19). These interactions result in activation of mitogen and stress-activated protein kinase (MAPK/SAPK) cascades, transcription factor activation, cytokine secretion, proliferation and differentiation of B cells into Ig-secreting plasma cells, and humoral memory establishment.

## Mechanism of Action

Up to date, there are three proposed mechanisms for the contribution of the CD40/CD40L dyad to T lymphocyte-dependent autoimmune diseases. The first proposed mechanism is mediated through improper T lymphocyte selection in the thymus. As CD40 has been shown to cooperate with the receptor activator of NF-κB (RANK) in promoting medullary thymic epithelial cells development, a disruption in CD40 activation in these cells could probably result in their inadequate development (20). Therefore, the disturbed development of medullary thymic epithelial cells potentially permits auto-reactive T lymphocyte clones to escape negative selection, leading to failure of central tolerance and the subsequent potential development of autoimmune diseases. The second proposed mechanism of CD40/CD40L contribution to autoimmune diseases occurs in secondary lymphoid organs, where T lymphocytes are primed by APCs (B lymphocytes or DCs) over-expressing CD40 either constitutively or transiently (20). The overexpression of CD40 leads to increased interactions between CD40L on T lymphocytes and CD40 on APCs, which favor the activation of auto-reactive T lymphocytes as well as the production of pro-inflammatory cytokines by antigen-presenting cells and auto-antibodies by B lymphocytes (21). Among the secreted pro-inflammatory cytokines, IL-6 has been shown to drive T lymphocytes differentiation into Th17 cells (21). In turn, Th17 cells induce cell-mediated tissue damage by secreting IL-17 (22). Therefore, the increase in CD40 expression and the subsequent exaggerated CD40/CD40L interactions potentially lead to the development of autoimmune diseases. The third proposed mechanism results from abnormal expression of CD40 in tissues where it is normally undetectable. Under such conditions, the tissues themselves contribute to the initiation of the autoimmune disease. Indeed, elevated expression levels of CD40 in target tissues (thyroid and pancreatic islet cells) have been associated with the initiation of thyroiditis and the production of inflammatory cytokines resulting in the failure of pancreatic islet cell transplants (23, 24). Otherwise, CD40/CD40L axis could be involved in the generation of Treg cells. Thus, the precise nature of the signals involving the generation of Treg cells has not been yet clarified, all we know is that Treg formation in the thymus is due to the involvement of TCR recognition of MHC-II expressed on thymic epithelium (25–27). However, a study published by Guiducci et al. demonstrated that there is a decrease in Treg number in the thymus of CD40^−/−^ mice, and of normal mice treated with Ab blocking CD40L, suggests that CD40/CD40L interaction takes part in the generation of Treg cells. Moreover, strong defects in survival and homeostatic proliferation were observed in CD40^−/−^ mice; however, the administration of exogenous IL-2 corrected these defects in CD40^−/−^ animals (28). Therefore, an overexpression of CD40/C40L could imply an increased generation of Treg cells promoting IBD since it is characterized by gut infiltration of highly reactive Treg cells (29).

**Figure 1:**
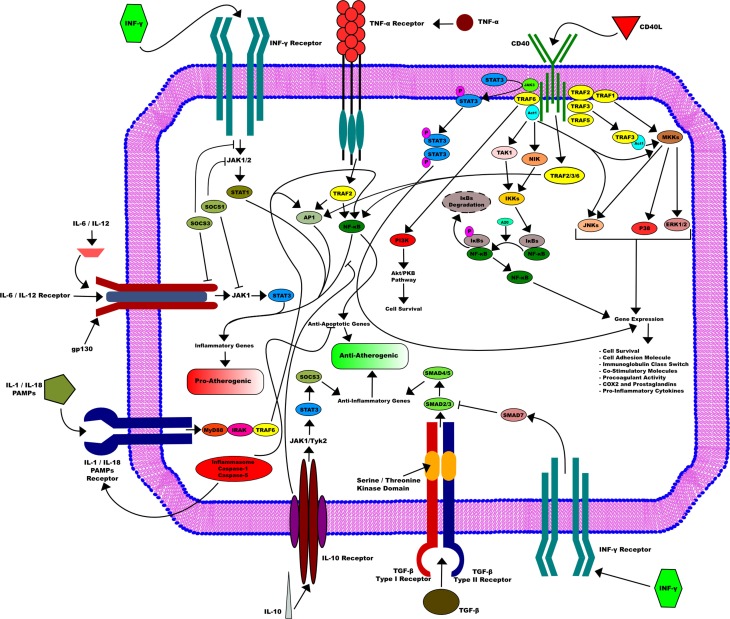
**CD40/CD40L axis in activated dendritic cells**. CD40/CD40L axis is implicated in many downstream pathways in activated dendritic cells that are essential for its function. CD40L induces the activation of dendritic cells through its binding to CD40. This activation requires the recruitment of the TRAFs family. This leads in turn to the activation of several pathways, such as STAT3, NF-κB, and MKKs. They are all mostly involved in many gene expressions that include cell survival, cell adhesion as well as procoagulant activity and pro-inflammatory cytokines production. On the other side, other pathways that go through different receptors influence DC activity, among them TNF-α inducing mostly an pro-atherogenic activity or IL-10 receptor that can enhance an opposite function and therefore promote an anti-atherogenic activity in DC.

Finally, we illustrated in Figure 1 the different pathways triggered by the axis CD40/CD40L in activated DCs.

## CD40/CD40L Pathway in Inflammatory Bowel Disease

The CD40/CD40L pathway activation is involved in the pathogenesis of IBD. Enhanced expression of CD40 and CD40L in the gut mucosa in IBD is reported (30–32). Furthermore, higher sCD40L levels in the circulation of IBD patients (33) as well as CD patients with abscesses and/or fistulas (34) were also pointed out, reflecting elevated surface expression and release of CD40L by activated platelets and activation of functional CD40L in the intestine.

The CD40/CD40L interaction was shown to be critical for the development of IBD through activation of various pathways related to inflammation in immune and non-immune cells. The initial evidences pointing the contribution of CD40/CD40L to the autoimmune IBD came from studies in mice. The results from these studies demonstrated that the CD40/CD40L interactions were an important element in the pathogenesis of colitis. In fact, administration of a blocking CD40L antibody at the onset of colitis initiation inhibits lymphocytic infiltration into the intestinal epithelium, and disease occurrence, whereas blocking CD40L 4 weeks following colitis initiation only improves the disease symptoms (35, 36).

Additionally, T lymphocytes CD40L overexpression in mice, results in T lymphocyte infiltration in multiple organs and death caused by IBD by 3–6 weeks of age (37). The particular relevance of this system in CD or UC is further emphasized by evidence obtained from IBD patients. CD40, CD40L, and sCD40L were observed to be strongly up-regulated in course of active CD and UC, particularly within the inflamed mucosa (30, 31, 33).

In patients with CD, CD40 is over-expressed on microvascular endothelial cells in the inflamed mucosa, along with an increase in CD40^+^ DCs found within the intestinal mucosa (38, 39). In contrast, genomic association studies did not show a correlation between the CD40 gene and IBD risk (40). Nonetheless, treatment of CD patients with the antagonist chimeric monoclonal anti-human CD40 antibody (ch5D12) showed a beneficial response and remission rates of 72 and 22%, respectively (41).

Several studies have shown that animal models of rheumatoid arthritis, experimental autoimmune encephalitis, and lupus nephritis yielded first remarks for the importance of CD40/CD40L pathways in immune diseases (42–44).

The CD40/CD40L axis was a source of curiosity in mouse models of IBD with a panoply of common pathogenic assets. Indeed, a study conducted *in vivo* showed the relevance of the CD40/CD40L system in the trinitrobenzene sulfonic acid (TNBS)-induced colitis (36).

Antibodies against CD40L have been effective in preventing the onset of the Th-1-driven colonic inflammation. This was due to an inhibited IL-12 production by antigen-presenting cells and the downstream lack of Th1 T cell priming. CD40L transgenic mice with high transgene copy numbers were predisposed to develop a lethal inflammation of the bowel. Moreover, mice showed a severe colitis with histopathological features of IBD. The diseased colon was marked by dense infiltrates of CD40L^+^CD4^+^ and CD8^+^ T cells and high numbers of CD40^+^ APCs (37). Thus, the data available from animal models strongly suggest that the CD40/CD40L system is a critical factor in the induction of inflammatory cascade in IBD and could then represent a target of treatment strategies.

**Figure 2:**
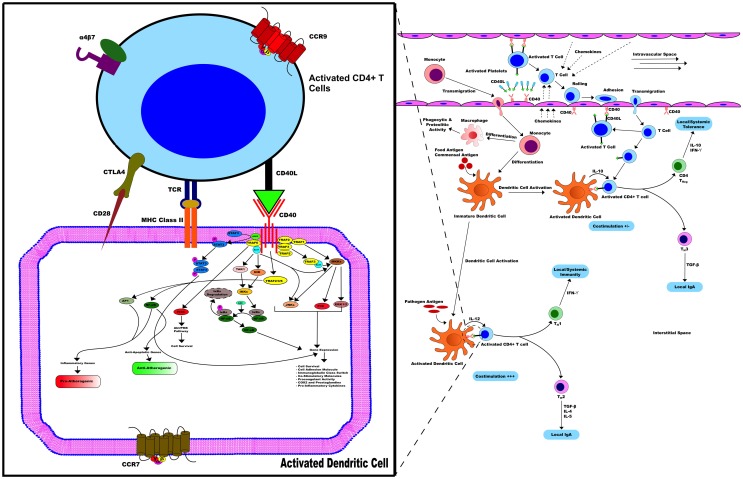
**CD40/CD40L in IBD**. CD40/CD40L axis contributes to the activation of various pathways related to inflammation in immune and non-immune cells, hence promoting IBD. In the early stages of mucosal inflammation, local T cells become activated and express CD40L, binding to and activating CD40^+^ DC. Therefore, CD40^+^ DC enhances cytokine secretion, such as IL-12, and up-regulation of co-stimulatory activity including CD40, CD40L, and MHC-II activity promoting more T cells that transmigrate into the interstitial space become activated with expressed CD40L. Activated T cells in the circulation of patients with IBD contribute to this process through expression of CD40L on their surface.

Furthermore, similar to multiple sclerosis and psoriasis, several genetic associations and disease-causing alleles have been identified for IBD (4, 40). Although none of the genomic loci associated with IBD incidence contain the CD40 gene (40), polymorphisms in genes related to the Th17 pathway including IL-12B, STAT3, and IL-23R confer increased risk of developing the disease (4, 40). CD40 signaling in multiple cell types leads to the production of IL-6, IL-12, and IL-23 and may, therefore, contribute to disease initiation and/or progression in susceptible individuals (21). All in all, Figure 2 summarizes all the events surrounding the potential implication of the axis CD40/CD40L in IBD.

This review presents the contribution of the CD40/CD40L axis in the pathogenesis of IBD. Better understanding of the pathogenesis of this condition represents the background for the progress in therapy. Data presented in this review are mainly based on physiological and pathological mechanisms. However, data from therapy approaches are highly relevant. Although the introduction of various biological agents designed to neutralize pro-inflammatory factors has been an important achievement toward the control of IBD, no curative treatment is currently available. In some animal models of colitis, anti-CD40L therapy was demonstrated to be effective (35). Indeed, this study demonstrated that administration of anti-CD40L to colitic mice induced significant clinical and histological improvement and down-regulated pro-inflammatory cytokine secretion. These data suggest that the CD40–CD40L interactions are essential for the Th1 inflammatory responses in the bowel in this experimental model of colitis. Therefore, in view of their critical role in the activation of antigen-presenting cells and T lymphocytes, targeting co-stimulating interactions of CD40/CD40L in IBD is a potential approach of antibody therapy. Thus, blockade of CD40 signaling may be beneficial to human IBD. However, further studies should be done in order to shed light on the importance of antibody therapy in the treatment of IBD.

## Author Contributions

NS, KK, and YZ contributed to literature search and writing of this review. CF and YD provided the two figures.

## Conflict of Interest Statement

The authors declare that the research was conducted in the absence of any commercial or financial relationships that could be construed as a potential conflict of interest.

## Abbreviations

APC, antigen-presenting cell; CD, Crohn’s disease; CD40, cluster of differentiation 40; CD40L, cluster of differentiation 40 ligand; DCs, dendritic cells; HIGM, X-linked hyper-IgM syndrome; IBD, inflammatory bowel disease; Ig, immunoglobulin; IFN-γ, gamma interferon; IL, interleukin; Th, T helper; TNFR, tumor necrosis factor receptor; TRAFs, TNFR-associated factors; UC, ulcerative colitis.
