# Supplementary material for: The Contribution of CD40/CD40L Axis in Inflammatory Bowel Disease: An Update
Source: Front Immunol. 2015 Oct 16;6:529. doi: 10.3389/fimmu.2015.00529 (PMC4607859; doi:10.3389/fimmu.2015.00529)
Supplement: Supplementary file 1 [file table_1.pdf]

**Table S1 | Cell types expressing the CD40/CD40L dyad**.

| Cell Type | CD40 | | CD40L | | Forms of CD40L |
|---|---|---|---|---|---|
| | Resting | Activated | Resting | Activated | |
| CD4$^+$ T lymphocytes | - | +++ | - | +++ | Membrane, soluble |
| B lymphocytes | +++ | +++ | ++ | +++ | Membrane |
| Macrophages | + | +++ | + | +++ | Membrane |
| Platelets | ++ | +++ | - | +++ | Membrane, soluble |
| Dendritic cells (DCs) | - | +++ | ++ | +++ | Membrane |
| Neutrophils | + | +++ | + | + | Membrane |
| Endothelial cells | + | +++ | + | + | Membrane |
| SMCs | +++ | N/A | + | N/A | Membrane |

*N/A, no documentation available; -, no expression; +, weak expression; ++, average expression; +++, strong expression.*
