# Supplementary material for: The Contribution of CD40/CD40L Axis in Inflammatory Bowel Disease: An Update
Source: Front Immunol. 2015 Oct 16;6:529. doi: 10.3389/fimmu.2015.00529 (PMC4607859; doi:10.3389/fimmu.2015.00529)
Supplement: Supplementary file 2 [file table_2.pdf]

Table S2 | Cell type specific CD40/CD40L-induced signaling.

CD40/CD40L axis	
**Cell type**	**Physiologic effect**
B lymphocyte	- Isotype switching<br>- Expression of MHC-I, MHC-II, VCAM-1, ICAM-1, LFA-1, FcεRII, B7.1, B7.2, and Fas<br>- Cytokine production (IL-6, IL-10, TNF-α, TGF- β, and LT-α)
Monocyte/ Macrophage	- Up-regulation of co-stimulory activity (ICAM-1, LFA-3, B7.1, and B7.2)<br>- Myeloperoxidase secretion<br>- Cytokine secretion (IL-1β, IL-6, IL-8, IL-12, and TNF-α)<br>- MMP secretion (MMP-1, MMP-2, MMP-3, and MMP-9)<br>- TF secretion
Platelet	- Platelet activation<br>- RANTES and ROS secretion
Dendritic cells (DCs)	- Cytokine secretion (IL-10 and IL-12)<br>- Up-regulation of co-stimulatory activity (CD40, CD40L, MHC-I, MHC-II, B7.1, and B7.2)
Neutrophil	ROS production - Enhanced Mac-1 expression
Endothelial cell	- Up-regulation of adhesion molecules (E-selectin, VCAM-1, and ICAM-1)<br>- Chemokines and cytokines secretion (IL-1, IL-6, IL-8, MCP-1, MIP-1α, and RANTES)<br>- ROS production - MMP secretion (MMP-1, MMP-3, and MMP-9) - Growth factor secretion (VEGF, FGF, and PAF)<br>- TF secretion<br>- Up-regulation of COX-2
SMC	Chemokines and cytokines secretion (IL-1β, IL- 6, IL-8, and MCP-1) - MMP secretion (MMP-1, MMP-3, and MMP-9) - TF secretion - Up-regulation of COX-2
T lymphocyte	CD8+ memory T lymphocyte generation
